# Supplementary material for: Patterns of Intron Gain and Loss in Fungi
Source: PLoS Biol. 2004 Nov 30;2(12):e422. doi: 10.1371/journal.pbio.0020422 (PMC532390; doi:10.1371/journal.pbio.0020422)
Supplement: Table S1 — Also available at http://genes.mit.edu/NielsenEtAl/. (4.3 MB ZIP). [file pbio.0020422.st001.zip › NielsenEtAl/html/681.html]

AN4727.1.NCU04442.1.MG08012.1.FG05689.1


```
 CLUSTAL W (1.82) Multiple Sequence Alignments - Introns Inserted


Sequence 1: MG08012.1	369 aa
Sequence 2: FG05689.1	377 aa
Sequence 3: NCU04442.1	375 aa
Sequence 4: AN4727.1	376 aa
Alignment Length: 382 aa
Number Identitical Residues: 243 aa
Alignment Score (without introns) 11064


MG08012.1 	MVRGTVLITG2GTGYIGSFTSLALLENDYDVVIVDNLYNSSAVAIDRIELICGKRPAFHN
NCU04442.1	MAVGTVLVTG2GTGYIGSFTTLALLDNGYDVVIVDSLLNSSKAALDRIELICGKRPTFYQ
FG05689.1 	MPVGTVLVTG2GTGYIGSFTSLTLLEHGYDVVIVDSLYNSSEVALDRIELICGRRPAFYK
AN4727.1  	MPSGSVLVTG2GTGYIGSFTTLALLEAGYKVVVADNLYNSSAEALNRIELISGKKAEFAQ
          	*  *:**:** *********:*:**: .*.**:.*.* ***  *::*****.*::. * :

MG08012.1 	VDITDEAALDKVFDAHPEIDSVIHFAALK~A----~-VGESGEIPLEYYRVNVGGSISLL
NCU04442.1	VDITDEAGLDDVFSKHPDIDSVIHFAALK0A----~-VGESGEIPLEYYRVNVGGSITLL
FG05689.1 	LDITDEKAIDEVFAKHPAIDSVIHFAALK~ASRIS0AVGESGEIPLEYYRVNVGGSISLL
AN4727.1  	LDVTDEAAFDKVFEAHPDIDSVIHFAALK0LYPLK~AVGESGEKPLDYYHVNVYGTICLL
          	:*:*** .:*.**  ** ***********     . :****** **:**:*** *:* **

MG08012.1 	RSMQKHNVCNIVFSSSATVYGDATRVPNMIPIPEHCPIGPTNTYGRTKSTIEDVISDHVN
NCU04442.1	RSMAKHNVSNIVFSSSATVYGDATRIPGMIPIPENCPIGPTNTYGHTKVMIETVISDHIE
FG05689.1 	RSMERNNVNNIVFSSSATVYGDATRFPNMIPIPEHCPIGPTNTYGRTKSMIEDVITDFVN
AN4727.1  	RSMVRHNVTNIVFSSSATVYGDATRFPDMIPIPEHCPLGPTNPYGNTKFAIELAITDVIN
          	*** ::** ****************.*.******:**:****.**.**  ** .*:* ::

MG08012.1 	AQRNNLKKAD--KPFDMWNGALLRYFNPCGAHPSGLMGEDPQGVPFNLLPLLGQVATGQR
NCU04442.1	AERRKAKKAG--KPFEQWNGALLRYFNPCGAHPSGIMGEDPQGVPYNLLPLLGKVATGER
FG05689.1 	AQRNNLEKAG--KEYKQWNGALLRYFNPCGAHPTGIMGEDPQGIPFNLLPLLGQVATGER
AN4727.1  	AQRNNAKKAGNETEAAKWNGALLRYFNPAGAHPSGIMGEDPQGVPYNLLPLLAQVATGKR
          	*:*.: :**....    ***********.****:*:*******:*:******.:****:*

MG08012.1 	EKLLVFGDD1YSSRDGTAIRDYIHVLDLAKGHLAALNYLRDNKPGVKAWNLGSGRGSTVF
NCU04442.1	DKLLVFGDD1YASKDGTAIRDYIHVLDLASGHLAALNYLREKKPGVKAWNLGSGRGSTVF
FG05689.1 	EKLLVFGDD1YSSRDGTAIRDYIHVVDLARGHLMALNYLRENQPGVKAWNLGSGRGSTVF
AN4727.1  	EKLLVFGDD1YASHDGTAIRDYIHILDLADGHLKALNYLRANNPGVRAWNLGTGRGSTVY
          	:******** *:*:**********::*** *** ****** ::***:*****:******:

MG08012.1 	EMIKAFSSVVGRDLPYEVVPRRQGDVLDLTANPALANKELGWKTELRMEDACQDLWKWVK
NCU04442.1	EMIKAFSKVVGRDLPYQVVDRRLGDVLDLTANPALANEELQWKTKLTLEDACADLWRWVE
FG05689.1 	EIINAFSKVVGRDLAYEVRPRRQGDVLDLTANPTLANKELNWKTELTMEEACNDLWRWVE
AN4727.1  	EMIRAFSKAVGRDLPYEVAPRRAGDVLNLTSNPTRANTELGWKAQRTLEQACEDLWLWTK
          	*:*.***..*****.*:*  ** ****:**:**: ** ** **::  :*:** *** *.:

MG08012.1 	NNPQGYRQDPPQEFVAALKK------
NCU04442.1	NNPKGYRQDPPAELLEKLKASKAVKN
FG05689.1 	NNPKGYRQDPPAKLLEAVKSSKA---
AN4727.1  	NNPQGYRQQPPAELLEQLKK------
          	***:****:** :::  :*
```
